# Intra- and Inter-Pandemic Variations of Antiviral, Antibiotics and Decongestants in Wastewater Treatment Plants and Receiving Rivers

**DOI:** 10.1371/journal.pone.0108621

**Published:** 2014-09-25

**Authors:** Andrew C. Singer, Josef D. Järhult, Roman Grabic, Ghazanfar A. Khan, Richard H. Lindberg, Ganna Fedorova, Jerker Fick, Michael J. Bowes, Björn Olsen, Hanna Söderström

**Affiliations:** 1 Natural Environment Research Council, Centre for Ecology and Hydrology, Wallingford, United Kingdom; 2 Section of Infectious Diseases, Department of Medical Sciences, Uppsala University, Uppsala, Sweden; 3 Department of Chemistry, Umeå University, Umeå, Sweden; 4 University of South Bohemia in Ceske Budejovice, Faculty of Fisheries and Protection of Waters, South Bohemian Research Center of Aquaculture and Biodiversity of Hydrocenoses, Vodnany, Czech Republic; 5 Section for Zoonotic Ecology and Epidemiology, School of Natural Sciences, Linnaeus University, Kalmar, Sweden; Gettysburg College, United States of America

## Abstract

The concentration of eleven antibiotics (trimethoprim, oxytetracycline, ciprofloxacin, azithromycin, cefotaxime, doxycycline, sulfamethoxazole, erythromycin, clarithromycin, ofloxacin, norfloxacin), three decongestants (naphazoline, oxymetazoline, xylometazoline) and the antiviral drug oseltamivir’s active metabolite, oseltamivir carboxylate (OC), were measured weekly at 21 locations within the River Thames catchment in England during the month of November 2009, the autumnal peak of the influenza A[H1N1]pdm09 pandemic. The aim was to quantify the pharmaceutical response to the pandemic and compare this to drug use during the late pandemic (March 2010) and the inter-pandemic periods (May 2011). A large and small wastewater treatment plant (WWTP) were sampled in November 2009 to understand the differential fate of the analytes in the two WWTPs prior to their entry in the receiving river and to estimate drug users using a wastewater epidemiology approach. Mean hourly OC concentrations in the small and large WWTP’s influent were 208 and 350 ng/L (max, 2070 and 550 ng/L, respectively). Erythromycin was the most concentrated antibiotic measured in Benson and Oxford WWTPs influent (max = 6,870 and 2,930 ng/L, respectively). Napthazoline and oxymetazoline were the most frequently detected and concentrated decongestant in the Benson WWTP influent (1650 and 67 ng/L) and effluent (696 and 307 ng/L), respectively, but were below detection in the Oxford WWTP. OC was found in 73% of November 2009’s weekly river samples (max = 193 ng/L), but only in 5% and 0% of the late- and inter-pandemic river samples, respectively. The mean river concentration of each antibiotic during the pandemic largely fell between 17–74 ng/L, with clarithromycin (max = 292 ng/L) and erythromycin (max = 448 ng/L) yielding the highest single measure. In general, the concentration and frequency of detecting antibiotics in the river increased during the pandemic. OC was uniquely well-suited for the wastewater epidemiology approach owing to its nature as a prodrug, recalcitrance and temporally- and spatially-resolved prescription statistics.

## Introduction

Pandemics are unique public health emergencies that can result in a large sudden increase in the use of a restricted set of pharmaceuticals within a short time period. In the case of an influenza pandemic, antiviral use will greatly exceed inter-pandemic use in most countries by several orders of magnitude, as few countries maintain significant inter-pandemic usage–Japan being a notable exception [1]. Depending on the severity of the pandemic, antibiotics have the potential to significantly exceed inter-pandemic usage for the treatment of secondary bacterial respiratory infections [2]. Decongestant usage is also predicted to increase with an increase in upper- and lower-respiratory tract infections [3].

Antibiotics, antivirals and decongestants are typically excreted as a large percentage of the parent dose in their bioactive form (mean: 82±22% for all drugs in this study (Table 1)) [4], [5]. The large load and high concentration of bioactive pharmaceuticals entering the wastewater and receiving rivers from widespread human consumption and excretion during a pandemic can potentially disrupt (micro)organisms through non-target effects [6]–[12] and cause the failure of wastewater treatment plants (WWTPs) to treat effluent to the required standard [13], [14], hasten the generation of antiviral resistance in wildfowl and other influenza-susceptible organisms [15]–[18], and accelerate the generation and spread of (novel) antibiotic resistance in the environment [2], [19], [20].

**Table 1:** Drug dosage, pharmacokinetics and limit of quantification (LOQ) of study analytes.

ATC Code	Drug	Class of pharmaceutical	ADQ^a^ (g)	% excreted^b^	LOQ (ng/L)
J01FA10	Azithromycin	Macrolide antibiotic	0.5	85%	1
J01DD01	Cefotaxime	Third-generation cephalosporin antibiotic	4	85%	10
J01MA02	Ciprofloxacin	Fluoroquinolone antibiotic	0.8	100%	5
J01FA09	Clarithromycin	Macrolide antibiotic	0.5	55%	4
J01AA02	Doxycycline	Tetracycline antibiotic	0.1	80%	1
J01FA01	Erythromycin	Macrolide antibiotic	1	100%	4
J01MA06	Norfloxacin	Fluoroquinolone antibiotic	0.8	90%	1
J01MA01	Ofloxacin	Fluoroquinolone antibiotic	0.4	98%	4
J01AA06	Oxytetracycline	Tetracycline antibiotic	1	35%	4
J01EC01	Sulfamethoxazole	Sulfonamide antibiotic	0.8	100%	2
J01EA01	Trimethoprim	Dihydrofolate reductase inhibitor	0.4	100%	1
R01AA08	Naphazoline	Vasoconstrictor decongestant	0.4	90%	1
R01AA05	Oxymetazoline	Vasoconstrictor decongestant	0.4	35%	7
R01AA07	Xylometazoline	Vasoconstrictor decongestant	0.8	90%	11
J05AH02	Oseltamivir carboxylate	Neuraminidase inhibitor antiviral	0.2	80%	2

a ADQ = is a measure of prescribing volume based upon prescribing behavior in England, which is similar, and often the same as the international standard defined daily dose (DDD).

b [4], [5].

**Figure 1:**
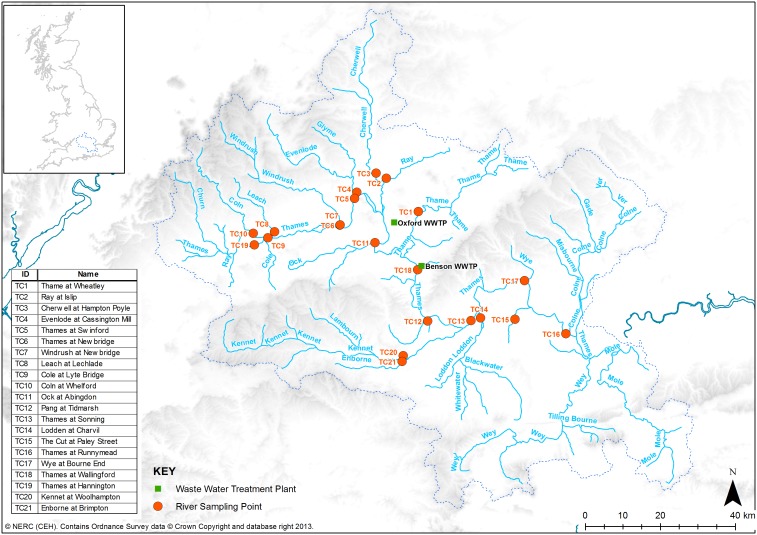
The 21 river sampling locations within the River Thames Catchment (TC) in southern England and the location of the Oxford and Benson WWTPs.

In this study, we measured eleven antibiotics, one antiviral and three decongestants (see Table 1) weekly at 21 locations within the River Thames catchment (Fig. 1) in England during the month of November 2009, the autumnal peak of the influenza A[H1N1]pdm09 pandemic. The aim was to quantify the pharmaceutical response to the pandemic and compare this to drug use during the late pandemic (March 2010) and the inter-pandemic periods (May 2011). One relatively large wastewater treatment plant (WWTP) in Oxford, UK employing activated sludge wastewater treatment and one relatively small WWTP in Benson, UK, employing trickle-bed wastewater treatment were sampled hourly for 24-h in November 2009 to 1) understand the differential pharmaceutical use patterns among the people within the WWTP catchments during the pandemic, 2) characterize the fate of the analytes in the two very different WWTPs prior to their entry in the receiving River Thames, and 3) examine the suitability of employing a wastewater epidemiology approach for the estimation of drug users within the Oxford and Benson WWTP populations.

## Experimental Section

### Wastewater Treatment Plant Characterisation

The Benson WWTP serves a population of 6,230 people with a consented dry weather flow (DWF) of 2,517 m^3^/d and an annual average DWF of 1,368 m^3^/d (Fig. 1 and Fig. S1 in File S1). The Benson WWTP has a hydraulic retention time of 7–8 h at DWF and consists of trickling filters as the main biological treatment step. Oxford WWTP serves a population of 208,000 with a consented DWF of 50,965 m^3^/d and an annual mean DWF of 38,000 m^3^/d (Fig. 1 and Fig. S1 in File S1). The Oxford WWTP has a hydraulic retention time of 15–18 h, and utilizes activated sludge as the main biological treatment step. Both WWTPs have primary and secondary sedimentation steps. Both Oxford and Benson WWTPs feed into the main stem of the River Thames (Fig. 1), separated by approximately 10 miles.

### Wastewater Treatment Plant Sampling

The sampling of all analytes (Table 1) in Benson and Oxford WWTP was performed during a 24-hour period spanning 10–11 November 2009. An additional 24-h sampling was initiated on May 11, 2011 from only the Benson WWTP effluent for the primary purpose of confirming the background concentration of the antiviral, oseltamivir carboxylate (OC), during the inter-pandemic period. The pandemic officially ended on August 10, 2010, hence, the expectation was that pharmaceutical use in the study catchments in May 2011 would reflect inter-pandemic pharmaceutical usage [21]. An automated sampler was used to recover time-proportional samples (approximately 750 ml) of influent and effluent every hour for 24 hours. Samples were aliquoted into triplicate 50-ml borosilicate glass vials with PTFE-lined caps and immediately stored at −80°C until analysis.

### River Sampling

Grab samples were acquired within 250 ml borosilicate brown glass bottles at the end of a 1.5 m-long sampling rod at 21 river locations within the River Thames catchment (Fig. 1). Sampling was undertaken on November 3, 11, 17, and 24, 2009, as well as on March 15, 2010 (late-pandemic period) and May 11, 2011 (inter-pandemic period). These sites are part of the CEH Thames Initiative Research Platform [22]. Samples were transported from the field to the laboratory within 6 hours and transferred into 50-ml borosilicate glass vials with PTFE-lined caps, in triplicate. The samples were stored at −80°C until analysis.

### River Flow

River flow data was acquired from the National River Flow Archive (http://www.ceh.ac.uk/data/nrfa/) for all locations at the closest gauging station to the sampling site (Fig. S2 in File S1). In the case of Loddon at Twyford (TC14) and The Cut at Binfield (TC15), the closest active gauging station was appreciably upstream. In these two cases we employed an infilling method (equipercentile transfer) to estimate the flow at the sampling location, as previously described [23].

### Environmental Conditions

Precipitation data for the 48 h before the sampling periods was used for the town of Benson, England (Fig. S3 in File S1) [24], which is geographically central to all river sampling locations and reflects the climatic conditions of all the sampling sites for the specific days of the study.

### Study analytes

Oseltamivir is a prodrug, which means it is metabolized *in vivo* to the active antiviral, oseltamivir carboxylate (OC). Eighty-percent of the parent dose is converted to OC, which is excreted and easily recorded in the environment. If only the parent compound was recorded one could not be sure that the drug wasn’t flushed down the drain prior to having been consumed or the result of improper disposal from a manufacturing plant [25]. Given that oseltamivir is not manufactured in the Thames catchment, the measures reflected in this study should reflect oseltamivir consumption and not improper disposal. The antibiotics examined in this study were selected as they reflect the drugs most likely to be used during an influenza pandemic [2], [26]. beta-Lactams were not included in this study owing to their relatively high propensity to hydrolysis and biodegradation and thus low likelihood for persistence in the environment. The decongestants examined in this study represent a cross section of this class of drug, and does not reflect all or even the majority of decongestants in use in England.

### Analytical Technique

An on-line solid phase liquid extraction/liquid chromatography-tandem mass-spectrometry (SPE/LC-MS/MS) method was used to measure the analyte levels in pre-filtered and acidified 1 mL-samples. This on-line SPE/LC-MS/MS method used has been evaluated and described in detail previously [27]. The on-line SPE/LC system consisted of a PAL HTC auto sampler (CTC Analytics AG, Zwingen, Switzerland), a Surveyor LC-Pump (Thermo Fisher Scientific, San Jose, CA, USA), an on-line SPE Hypersil GOLD C18 column (20 mm×2.1 mm i.d.×12 µm, Thermo Fisher Scientific, Waltham, MA, USA), an Accela LC pump (Thermo Fisher Scientific, San Jose, CA, USA), and a Hypersil GOLD C18 column (50 mm×2.1 mm i.d.×3 µm particles, Thermo Fisher Scientific, San Jose, CA, USA) with a guard C18 column (2 mm×2 mm i.d.×3 µm particles, Thermo Fisher Scientific, San Jose, CA, USA). The liquid chromatography system was coupled to a heated electrospray ionization (HESI) source and a Quantum Ultra triple quadrupole mass spectrometer made by Thermo Fisher Scientific (Waltham, MA). The MS/MS parameters used are described in Table S3 in File S1. The following internal standards were obtained from Cambridge Isotope Laboratories (Andover, MA, USA): ^13^C_2_-Trimethoprim (^13^C_2_-TRI) (99%), ^13^C_3_15N–Ciprofloxacin (^13^C_3_-CIP) (99%), ^13^C_2_-Erythromycin (^13^C_2_-ERY) and ^13^C_6_-Sulphamethoxazole (^13^C_6_-SUL). Oseltamivir carboxylate labeled with deuterium (OCD3) (RO0604802-004; lot: 511-001-2197/4) was obtained from Roche (F. Hoffmann-La Roche Ltd., Basel, Switzerland).

### Fate in the WWTPs and the Thames River Catchment-Calculating WWTP and River Load and Percent Loss in WWTP

Pharmaceutical concentrations were converted to mass loading using hourly WWTP (Fig. S1 in File S1) and river flows for the sampling period. Percent loss of analytes between WWTP influent and effluent as a result of biodegradation and sorption was calculated from the change in 24-h load of each analyte between the two sampling locations. As the influent and effluent samples were acquired simultaneously, the calculated ‘percent loss’ assumes negligible change in drug use between the Monday and Tuesday during which the samples were acquired and a negligible change in hydraulic retention time. Resulting from such assumptions, the interpretations of the recalcitrance of analytes were considered with caution.

### Wastewater Epidemiology-Forward-Calculating Environmental Concentrations of Antibiotics from Prescription Statistics

The National Health Service Business Services Authority (NHS BSA) annual antibiotic prescriptions for England [28] was used for estimating ‘background’ pharmaceutical use for the population residing within the two study WWTP catchments in 2009. NHS BSA data are resolved at the national level and reflect annual prescription rates (Table S1 in File S1). More spatially resolved data was acquired from the four Primary Care Trust (PCT) [29] hospitals and clinics serving the Oxford and Benson WWTP catchments, however, this level of detail was only available from November 2011. To assess the value of this data as a proxy for ‘background’ antibiotic use in November, we examined total antibacterial use in general practice in England since 2007. There was a <1% change in total antibiotic prescriptions per year with the exception of penicillin where an increase of approximately 5% was seen between 2007–2011 [28]. As penicillins were not monitored in this study, we argue that the November 2011 data might serve as an adequate proxy for ‘background’ antibiotics prescribed during the study period. A large fluctuation from this ‘background’ usage might be indicative of pandemic-linked usage.

The National Pandemic Flu Service (NPFS) [30] recorded approximately 66,218 courses of Oseltamivir dispensed in Week 43 in 2009, representing 0.13% of the population of England, and 6% of all antivirals dispensed during the pandemic [30]. The national peak for the autumnal wave of the influenza pandemic was 3 weeks prior to the WWTP sampling on 10–11 November [30]. The antiviral prescription rate did not rapidly decline after the peak (see Fig. 15 in [30]), suggesting that the peak antiviral prescription rate of 0.13% might be a good proxy for antiviral use during the sampling period. The standard adult Oseltamivir dosing regime was assumed: 0.075 g per dose, consumed twice per day (0.150 g/d).

An additional dataset produced by the HPA’s QSurveillance National Syndromic Surveillance System [31], was examined for estimating Oseltamivir prescription rates. The HPA dataset reports 54.2 people per 100,000 with influenza-like illness (ILI) in the Oxfordshire PCT during the week of WWTP sampling (i.e., Week 46), which was used for modelling purposes. However, the ILI reporting rate declined during November from Week 46 to Week 49, which reached as low as 33 per 100,000 [31].

We provide a general model for calculating the concentration of pharmaceuticals (ng/L) in wastewater influent (C_w_) using the different data sources discussed above:
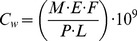
(1)where the product of the population of each WWTP catchment (P) and the volume of wastewater per person (*L*; 230 L/capita/d [2]) was divided into the product of the mass of prescriptions (*M*) in grams acquired from Average Daily Quantity (ADQ) conversions (Table 1 and S1) [32], mass of parent compound excreted in its parent form (*E*) in grams, and correction factor (*F*) for adjusting for when the population served by a PCT is served by more than one WWTP.

When deriving the mass of drug prescribed using NHS BSA statistics, *M* = *M_a_+M_s_*, where M_a_ was the annual mass (g) of pharmaceutical prescribed (Table S1 in File S1) and M_s_ reflected the additional mass of drug used (g) in the winter in excess of the average monthly usage (Table S1 in File S1). Hence, M_s_ = (M_a_×0.09375), where 0.9375 is the additional fraction of drug used in the winter as compared to the annual mean (i.e., M_a_/12). This adjustment was performed because it is known that the mean variation in antibiotic consumption between the summer and winter period in the UK was approximately 18.75% in 2005 [33]. Hence, the annual prescription rate provided by the NHS BSA was increased by 9.375% from the annual mean prescription rate (i.e., reflecting 50% of the total seasonal variability between summer and winter). Differences in pharmacokinetics were accounted for using factor *E*, which reflects the fraction of parent chemical excreted into wastewater (Table 1) [4].

The patient population of the PCT serving Benson is served by two different WWTPs at Benson and Cholsey. This difference between the population served by a PCT and the WWTP catchment were accounted for by factor *F*, where *F* = 0.389, resulting from the ratio of the population served by the Benson WWTP (6230) to the patient population of the local PCT (16,000). However, *F* = 1 for the Oxford WWTP as it was assumed that the populations served by the PCT in Oxford all fed into the Oxford WWTP. Lastly, *F* = 1 for all NHSBSA statistics, as the NHSBSA dataset used a national average prescription rate and was not stratified to the local level.

Notably, trimethoprim and sulfamethoxazole are routinely dispensed as a mixture, co-trimoxazole. Use of these drugs was calculated assuming one ADQ of co-trimoxazole contained 0.16 g of trimethoprim and 0.8 g of sulfamethoxazole.

All raw data has been made freely available at http://doi.org/10/t2x.

## Results

### PharmaceuticalFate in Benson and Oxford WWTPs

#### Antiviral in Benson WWTP

The concentration of OC in the influent on 10–11 November, 2009, ranged from <limit of quantification (LOQ; 1–11 ng/L, see Table 1) to 2070 ng/L (Table 2), with 18 of the 24 measures above the LOQ (Fig. S4 in File S1). The mean hourly concentration (for samples >LOQ), was 433±472 ng/L. The 24-h load was 410 mg/d, reaching a maximum load of 133 mg/h at the 18∶00 sampling point, equating to 66 µg OC/capita/d.

**Table 2:** Mean concentration and load of pharmaceuticals measured within the Benson and Oxford WWTP on November 10–11, 2009 (n = 24).

		Oxford			Benson		
		Load (mg/d)			Load (mg/d)		
		Mean concentration (ng/L)^a^			Mean concentration (ng/L)		
		Inlet	Outlet	% Loss^b^	Inlet	Outlet	% Loss
Antibiotic							
		4780	492		32.3	21.8	
	Azithromycin	163±96	30±6	90%	40±20	34±10	32%
			116		6.80		
	Cefotaxime	<LOQ	51	<	18±7	<LOQ	>99%
		57300	1840		221	1.2	
	Ciprofloxacin	1090±300	52±50	97%	200±303	14±3	99%
		27800	2590		43.7	27.3	
	Clarithromycin	524±179	92±27	91%	61±62	50±21	38%
		3230	6270		414	57.9	
	Doxycycline	60±43	121±83	–94%	345±444	67±150	86%
		69500	9440		847	234	
	Erythromycin	1330±560	236±40	86%	954±1610	244±83	72%
		9680	332		37	13.6	
	Norfloxacin	184±80	21±1	97%	59±26	25±5	63%
		4260	472		1462	9.68	
	Ofloxacin	81±26	23±8	89%	2320±3340	195	99%
		56100	697		236	51.3	
	Oxytetracycline	1090±229	29±16	99%	171±400	174±83	78%
		8960	2970		75.6	32.7	
	Sulfamethoxazole	169±28	67±18	67%	57±118	34±6	57%
		3550	3840		312	49.0	
	Trimetoprim	70±14	73±7	–8%	332±190	68±66	84%
Decongestant							
					1460	69.2	
	Naphazoline	<LOQ	<LOQ	na	1650±775	696±981	95%
			65.8		43	172	
	Oxymetazoline	<LOQ	16±1	na	67±61	307±110	–299%
			28.0		6.82		
	Xylometazoline	<LOQ	15	na	13±11	<LOQ	na
Antiviral							
		18600	18900		410	206	
	Oseltamivir	350±59	358±60	–2%	433±472	208±40	50%

Percent loss calculated from change in daily pharmaceutical load between the influent and effluent.

a Mean concentrations are followed by standard deviation for all samples >LOQ during the 24-hour sampling.

b Negative values indicate a concentration increase from inlet to outlet. ‘<’ is used to indicate concentration where the influent was below the LOQ while the effluent was >LOQ. na = not applicable.

The concentration of OC in the effluent ranged from <LOQ to 287 ng/L, with 21 of the 24 measures above the LOQ. The mean hourly concentration (for samples >LOQ), was 208±40 ng/L. The 24-h load was 206 mg/d, reaching a maximum of 16.9 mg/h at the 9∶00 sampling point. The change in load from the influent to the effluent was 204 mg/d, a reduction of 50%.

#### Antiviral in Oxford WWTP

The concentration of OC in the influent on 10–11 November, 2009, ranged from 257 to 550 ng/L (Table 2), with all measures above the LOQ. The mean hourly concentration was 350±59 ng/L. The 24-h load was 18,600 mg/d, equating to 89 µg OC/capita/d reaching a maximum load of 1,330 mg/h at the 10∶00 sampling point.

The concentration of OC in the effluent ranged from 474 to 1,130 ng/L, with all measures above the LOQ. The mean hourly concentration was 358±60 ng/L. The 24-h load in the effluent was 18,900 mg/d, reaching a maximum of 1130 mg/h at the 8∶00 sampling point. The change in 24-h load from the influent to the effluent was −300 mg/d, a trivial increase of 2% in the effluent, suggestive of a fully conservative chemical.

#### Antibiotics in Benson WWTP

Erythromycin showed the highest average antibiotic concentration in the influent, 954 ng/L (n = 17, 134 µg/capita/d) and effluent 244 ng/L (n = 20, 37 µg/capita/d; Table 2) and reached as high in concentration as 6,870 ng/L in the influent. Three antibiotics, at least once in the 24-h sampling, exceeded the average inlet concentration of erythromycin: ofloxacin (max = 11,000 ng/L, 235 µg/cap/d, n = 12), doxycycline (max = 1,550 ng/L, 66 µg/cap/d, n = 23) and oxytetracycline (max = 1,700 ng/L, 38 µg/cap/d, n = 20). Notably, ciprofloxacin was recorded as high as 917 ng/L (35 µg/cap/d, n = 16) and trimethoprim as high as 780 ng/L (50 µg/cap/d, n = 21).

With the exception of cefotaxime, which was found in only 7 of 24 samples from Benson influent, all antibiotics were found in at least half of the 24 samples, with 6 antibiotics found at each of the 24 hourly measurements (Fig. S4 in File S1).

The load of each of the 11 antibiotics in the Benson WWTP effluent was reduced by 32% to a maximum of 99%+ after treatment (Table 2), the most persistent being azithromycin (32% loss, n = 18 and n = 15 for inlet and outlet, respectively) and clarithromycin (38% loss, n = 13 and n = 11, respectively).

#### Antibiotics in Oxford WWTP

Erythromycin showed the highest average antibiotic concentration in the influent (1,330 ng/L, 341 µg/cap/d, n = 24) and reached a maximum concentration of 2,930 ng/L (Table 2). Two other antibiotics recorded mean concentrations in the influent above 1,000 ng/L, oxytetracycline (1,090 ng/L, 275 µg/cap/d, n = 24) and ciprofloxacin (1,090 ng/L, 281 µg/cap/d, n = 24). Two antibiotics achieved maximum concentration that exceeded the average inlet concentration set by erythromycin: oxytetracycline (1,430 ng/L) and ciprofloxacin (1,530 ng/L; Table 2). Notably, clarithromycin achieved a maximum of 980 ng/L (136 µg/cap/d), much higher than the maximum in Benson (243 ng/L, 7 µg/cap/d).

With the exception of cefotaxime (0/24), azithromycin (13/24) and trimethoprim (23/24), all antibiotics were found at each of the 24 hourly measurements from the Oxford influent.

The antibiotic load in the Oxford WWTP effluent was reduced by 67% to <LOD after treatment for many antibiotics (Table 2), however, unlike Benson, several demonstrated persistence and concentration, including: cefotaxime (increase from <LOQ to 51 ng/L (n = 1) for inlet and outlet, respectively), doxycycline (increase by 94% from 3230 ng/L (n = 24) to 6270 ng/L (n = 24), respectively) and trimethoprim (increase by 8% from 70 ng/L (n = 24) to 73 ng/L (n = 24), respectively).

#### Decongestants in Benson WWTP

Approximately 1.56 g/d of decongestant was quantified in the Benson WWTP influent (0.25 mg/capita/d), 97% of which was a single decongestant, naphazoline. The most frequently quantified decongestant in the influent was naphazoline (n = 19), however, oxymetazoline was the most frequently found decongestant in the effluent (n = 18; Table 2). Oxymetazoline achieved a mean influent and effluent concentration of 67 ng/L and 307 ng/L, respectively. Unlike oxymetazoline, naphazoline averaged higher concentrations in the influent (1,650 ng/L) than the effluent (696 ng/L). Naphazoline reached a higher maximum concentration in the influent and effluent (3,070 ng/L, 1,390 ng/L, respectively) than oxymetazoline (177 ng/L, 440 ng/L, respectively). The influent load (1,460 mg/d) for naphazoline, was reduced by 95% as compared to the effluent (69 mg/d, n = 2), indicating it is unlikely to be a persistent environmental pollutant. Xylometazoline averaged concentrations only marginally above the LOQ (11 ng/L) in the influent (13 ng/L; 7 mg/d, n = 10), and was <LOQ in all of the effluent samples.

#### Decongestants in Oxford WWTP

Unlike at Benson WWTP, no decongestant was found from the Oxford WWTP influent, within the limits of quantification. However, decongestants were quantified from the Oxford WWTP effluent during the hours 2∶00 and 3∶00–notably the time when the Oxford WWTP influent flow was at its minimum (Fig. S1 in File S1); these were xylometazoline (15 ng/L, 28 mg/d, n = 1) and oxymetazoline (16 ng/L, 66 mg/d, n = 2), the latter of which indicated a propensity for concentration within the Benson WWTP, while the former did not.

### Pharmaceutical Occurrence in the Thames River Catchment

#### Antiviral

**Figure 2:**
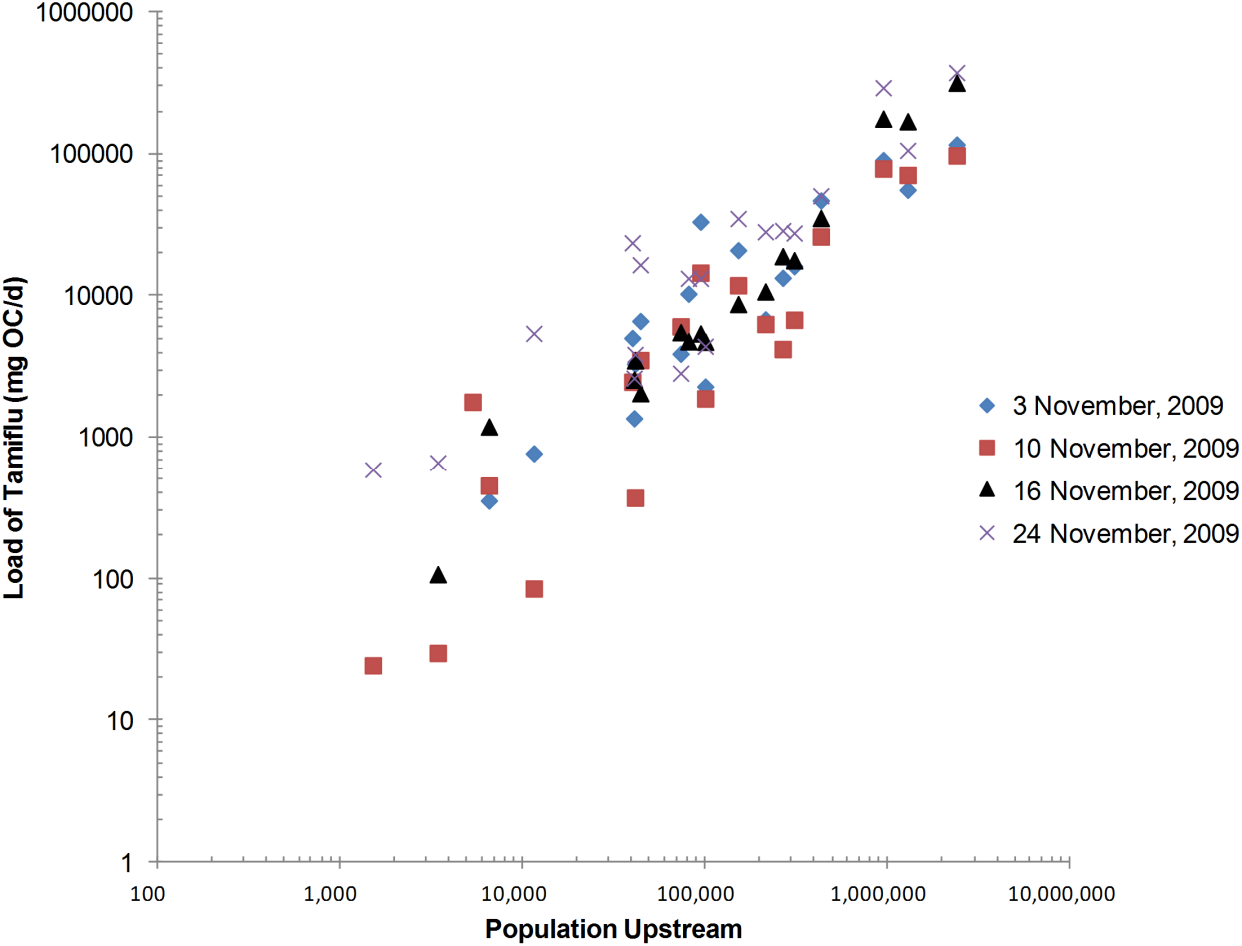
Correlation between population upstream and daily load of OC in river (mg OC/d) for each of the four sampling points: November 3 (diamond), November 10 (square), November 16 (triangle) and November 24 (‘x’), 2009.

**Figure 3:**
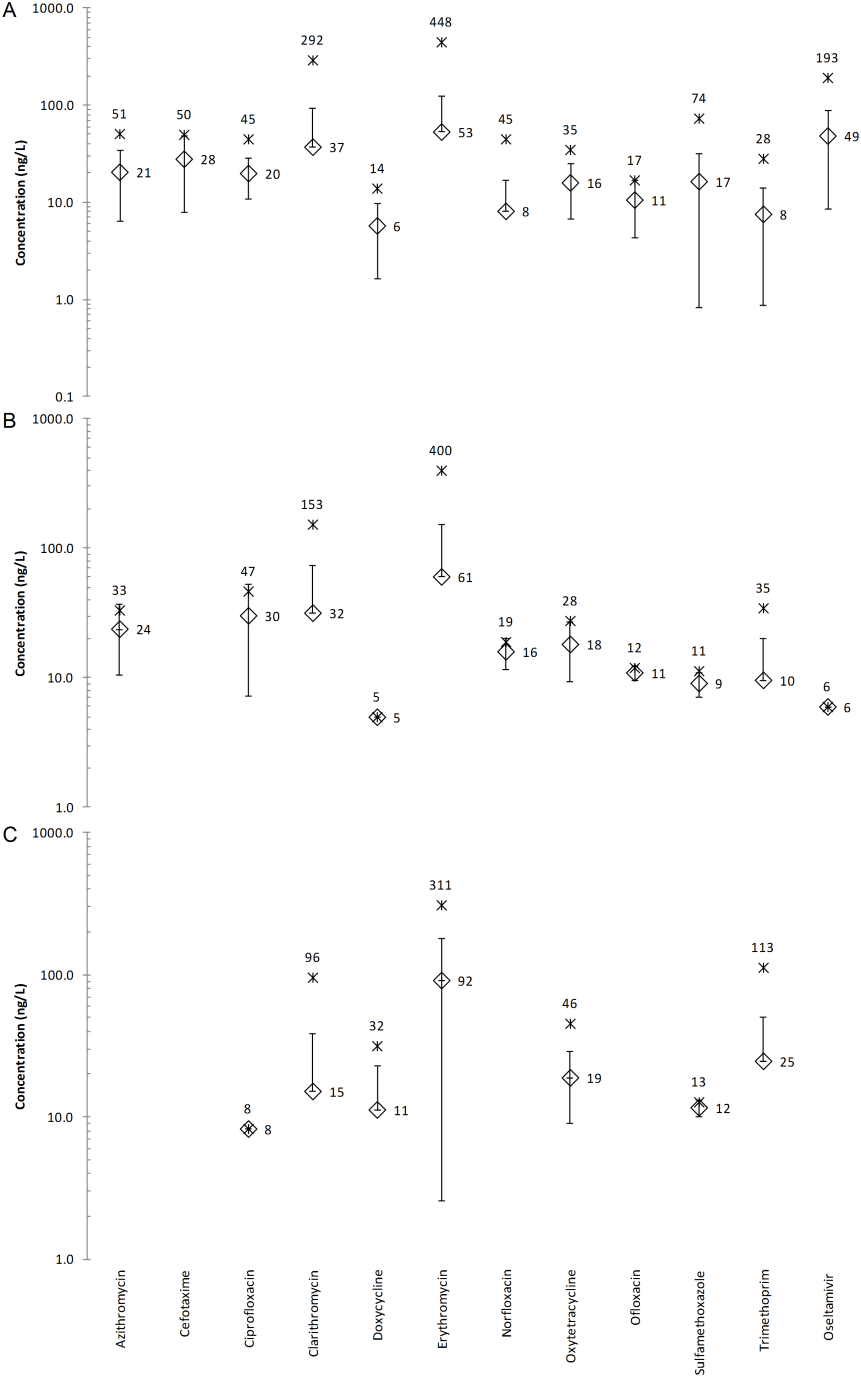
Mean (diamond; with standard deviation (upper and lower error bar)) and maximum (asterisk) concentration (ng/L) of pharmaceuticals across the 21 River Thames locations during (A) the four sampling occasions in November 2009 (intra-pandemic, n = 84); (B) 15 March, 2010 (late-pandemic. n = 21); and (C) 11 May, 2011 (inter-pandemic, n = 21). ‘Maximum’ data label is above asterisk, while ‘mean’ data label is to the right of the diamond. Decongestants were omitted as none were found >LOQ.

**Figure 4:**
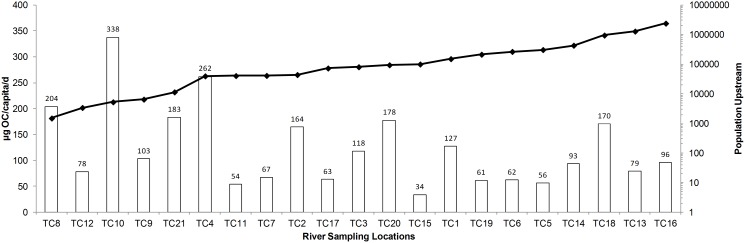
Mean load of oseltamivir (OC) per capita per day (µg/cap/d) across all river sampling locations throughout November 2009 (intra-pandemic period). Triangles indicate the population upstream at that location.

OC was the most frequently measured analyte with concentrations >LOQ at 73% of the river sampling locations during the month of November, 2009. The mean concentration of OC across the Thames catchment was 65, 61, 33 and 33 ng/L for November 3, 10, 16, and 24, respectively (Fig. 2 & 3). A maximum OC concentration of 193 ng/L was recorded at The Cut at Paley Street (T15) on Nov 10 (Figs. 3, S5b in File S1), a site known to be among the more severely impacted by sewage [22] with relatively low dilution per capita (Fig. S2 in File S1). The mean load of OC across all 21 sites was 24, 18, 46 and 55 g OC/d for November 3, 10, 16 and 24, respectively, with a maximum load on November 24 at the Thames at Runnymead (TC16) site, the location with the highest upstream population (2.3 million; Fig. S2 in File S1). A strong positive correlation (R^2^ = 0.82 to 0.96) between the population upstream of a sampling location and the load of OC further confirms this expected relationship (Fig. 2). The Thames at Runnymead (TC16; Fig. 1), recorded 117, 98, 319, and 377 g OC/d (49, 41, 134, 158 µg/capita/d, respectively) for the same time points in November (Fig. 4). With only a few exceptions, the per capita usage at TC16 (the most downstream sampling point on the River Thames) was consistent with estimates generated from many of the upstream sampling sites (Fig. 4), indicating that OC was relatively conserved within the river environment.

OC was not found in any river sampling location in March 2010 or May 2011 except for one location in March, TC17 (River Wye at Bourne End; Fig. S5e, f in File S1). In this location a concentration of 6 ng/L was recorded with an estimated load/d of 622 mg/d or 8.48 µg/capita/d, approximately 13% that achieved during the month of November (63 µg/capita/d) at this site.

#### Antibiotics

**Figure 5:**
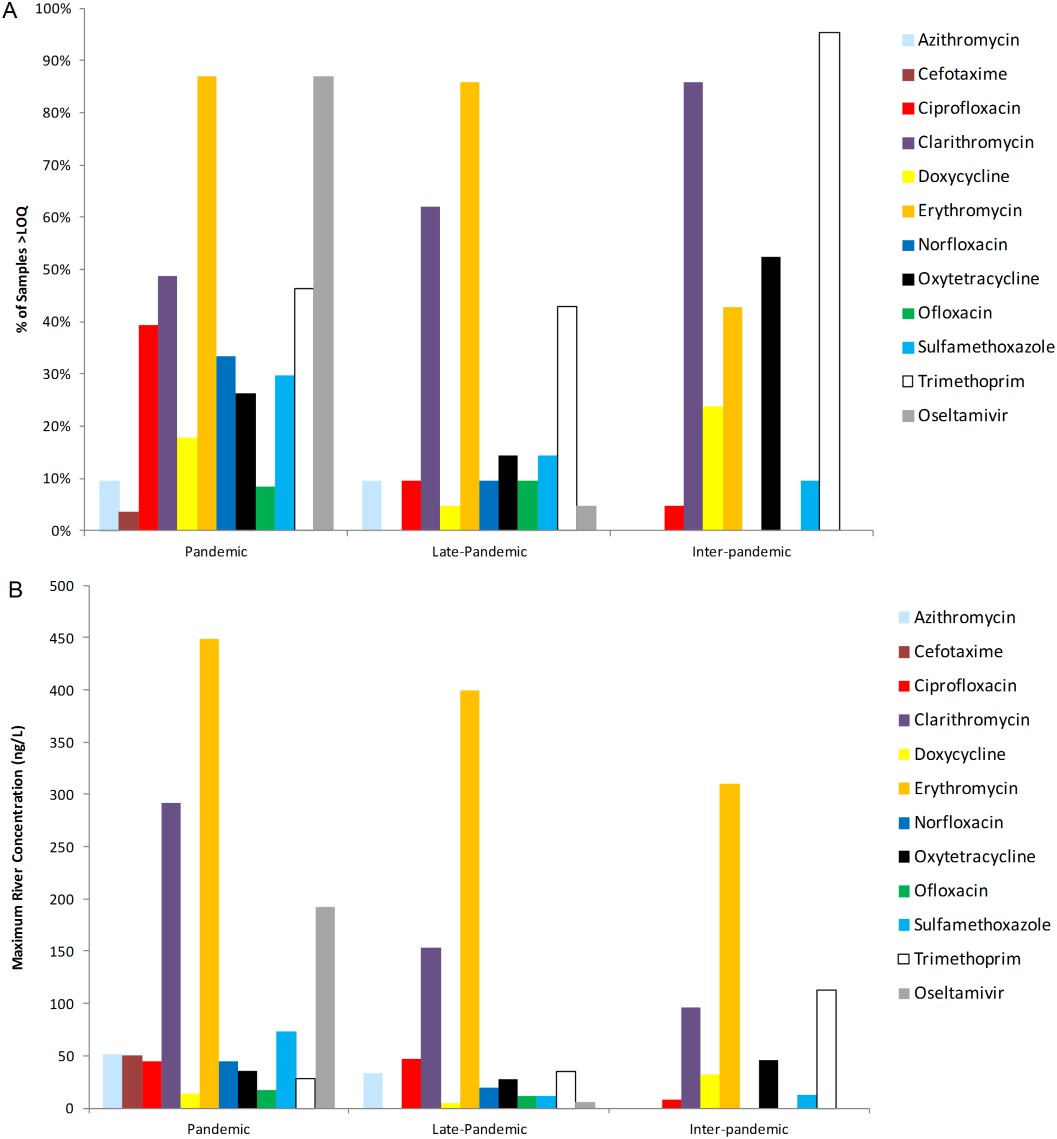
Comparison of pharmaceutical (A) abundance (% of samples above the limit of quantification (LOQ)) and (B) the maximum river concentration (ng/L) during the pandemic period (November, 2009), late pandemic period (March 2010) and the inter-pandemic period (May 2011).

The concentration range for antibiotics largely fell within the low-ng/L range (17–74 ng/L; Figs. 3, S5a–e in File S1), with the exception of clarithromycin (max = 292 ng/L) and erythromycin (max = 448 ng/L), at TC15 and TC14, respectively. Much like OC, the load of antibiotics across all time points was positively correlated with the number of people upstream of the sampling location. Erythromycin was by far the most frequently recorded antibiotic, >LOQ in 87% of samples, equally as abundant as OC (Fig. 3 & 5). Clarithromycin and trimethoprim were recorded in approximately 50% of samples. Ciprofloxacin and norfloxacin were measured in 39 and 33% of samples. Sulfamethoxazole was found in 30% of the river samples, while they were recorded in 79–100% of WWTP inlet and 68–83% of outlet samples. Similarly, doxycycline was frequently found in WWTP inlet and outlet samples (96–100% and 71–100%, respectively), but only measured >LOQ in 18% of river samples.

The mean load of antibiotics (µg) per capita (upstream population) per day was calculated for each of the analytes across the four November sampling times. Estimates range between 84 µg/cap/d for TC6, where only 4 antibiotics were recorded (ciprofloxacin, clarithromycin, erythromycin and norfloxacin), to 893 µg/cap/d for TC4 where six antibiotics were recorded. Despite all 11 antibiotics being recorded at TC15, it was among the lower yielding sites at 119 µg/cap/d. The low levels of antibiotics per person in The Cut at Paley Street could be down to the fact that The Cut is impacted by a very small number (3) of relatively large WWTPs (Bracknell = 77600 person equivalents (PE); Ascot = 26000 PE; White Waltham = 5150 PE), and therefore all the effluent has a relatively good level of treatment. There are no small WWTPs in this catchment, unlike all the other sites. The Cut was also one of only three sites to record cefotaxime. The sites that reflect the highest population upstream, TC18, TC13 and TC16, yielded estimates of 633, 199 and 263 µg/cap/d and recorded 8 or 9 of the 11 antibiotics.

#### Late-pandemic/Inter-pandemic period

Fewer antibiotics were found at the late-pandemic period (mean 2.6±2.6; March 15, 2010) and inter-pandemic period (mean 3.1±1.7; May 11, 2011) than in the pandemic period (mean 6.9±2.3; Figs. 5 and S5a–e in File S1). The load of antibiotic found at each river location during the inter-pandemic period (May 2011) was less than that found during the pandemic (Figs. S5a–e in File S1). In all but three cases (TC11, TC15 and TC17), the same was true for the late-pandemic sampling points. Notably during the late-pandemic period, erythromycin was found at a concentration of 400 ng/L in TC15, a highly sewage impacted site; a site that achieved nearly 750 ng/L in total antibiotics. The high erythromycin level was again found in the inter-pandemic sampling (May, 2011) at TC17, achieving 311 ng/L (Fig. S5e in File S1). At site TC15, the level of trimethoprim was also exceptionally high (113 ng/L), far exceeding the maximum concentration found during the pandemic (November, 2009; 28 ng/L). It is unclear whether these levels are the result of a local outbreak, normal fluctuation, or evidence of improper disposal.

#### Decongestants

Naphazoline, the most frequently found decongestant in the Benson WWTP was not found in the river samples. Oxymetazoline in the Benson WWTP effluent, along with xylometazoline, was also not found in the river samples. Decongestants were also not found in the river samples collected during the late-pandemic or the inter-pandemic period.

### Drug Use by Wastewater Epidemiology

Estimation of oseltamivir compliance, i.e., use of the drug as prescribed, was approximately 45–60% as previously reported [34]. Estimation of antibiotic use were derived from government statistics (mg/excreted/d; Table 3). Overall, neither of the government statistics (PCT or NHS BSA) were accurate predictors of MELs.

**Table 3:** Comparison of measured environmental load (MEL) of antibiotics in WWTP influent to MEL estimates generated from PCT- and NHS BSA-derived prescriptions.

	Benson			Oxford		
	mg/excreted/d^a^		mg/d	mg/excreted/d^a^		mg/d
	PCT^b^	NHS BSA^c^	MEL^d^	PCT	NHS BSA	MEL
Azithromycin	55.2	75.7	32.3	652	6493	4782
Cefotaxime	na	na	7.00	na	na	<LOQ
Ciprofloxacin	389	1229	221	7150	105409	57258
Clarithromycin	89.2	441	43.7	2035	37810	27846
Doxycycline	54.0	34.2	414	757	2933	3234
Erythromycin	1259	3920	847	18833	336086	69475
Norfloxacin	0	0	37	0	0	9677
Ofloxacin	0	6	1462	26.1	479	4257
Oxytetracycline	195	187	236	3080	16040	56136
Sulfamethoxazole	51.9	103	75.6	693	8799	8956
Trimethoprim	800	1120	312	6693	96060	3548

a Predicted amount of drug excreted from the daily doses (ADQ) of antibiotic consumed during 24-h sampling from Benson and Oxford WWTP influent as per: ^b^ Primary Care Trust (PCT) and ^c^ National (NHS BSA) statistics for antibiotic use.^ d^ Measured environmental load (MEL) from 24-hourly wastewater inlet samples (sum of each antibiotic/24 h). No biodegradation other than pharmacokinetics (Table 1) was assumed. na = prescription statistics were unavailable.

## Discussion

### Pharmaceutical Fate and Occurrence in Oxford and Benson WWTPs, and the Thames River Catchment

#### Antiviral

Mean concentrations of OC in the WWTP influent (350, 443 ng/L) and effluent (208, 358 ng/L) were consistent with the range previously reported in the literature 11–1450 ng/L (Table S2 in File S1) [1], [25], [35]–[39]. The maximum concentration found in this study (2,070 ng/L, Benson influent) was the highest reported in the literature during the pandemic (827 ng/L [35]). The persistence of OC in the Oxford WWTP is consistent with many reports in the literature that document minimal loss of OC in laboratory and field studies [13], [35], [37], [38], [40]. However, the extent of OC loss seen in the Benson WWTP (approximately 50%), was well above the range reported for WWTP not using ozonation, but it is consistent with the removal efficiency reported in WWTPs with ozonation in Germany, where elimination of OC was reported to be 59% [25], and Japan, where loss was between 30–40% [38].

Given the mild nature of the pandemic, measured environmental concentrations (MECs) of OC (33–62 ng/L) were 2–3 orders of magnitude lower than predicted environmental concentrations (PECs) in this catchment during a severe pandemic, i.e., R_0_>2.0 [2], [7], but is consistent with the lower end of PECs for a mild pandemic within the Thames catchment (27–11,000 ng/L; [2]). As expected, no OC was found during the inter-pandemic period (May 2011) from either the WWTP effluent or river sampling locations, reinforcing the assumption that Oseltamivir was used in negligible amounts during the inter-pandemic period in the UK (Fig. 5).

Previous studies have demonstrated OC resistance development where influenza-infected mallard ducks had been exposed to water containing OC at concentrations ≥1000 ng/L [15], [18], which closely approximates concentrations recorded in Japan [36], [41] and within an order of magnitude of many measured concentrations in the literature and this study (Table S2 in File S1). The evidence in this study and the literature further lend support to the likelihood of OC-resistance generation in wildfowl influenza viruses; owing to its significance to human health, this is worthy of additional study.

#### Antibiotics

In general, the antibiotics were more labile in the Oxford WWTP as compared with Benson, mostly likely due to the fact that Oxford uses activated sludge treatment while Benson relies on a trickling filter to treat wastewater. Fewer different antibiotics were recovered in the river during the late-pandemic (March 15, 2010) and the inter-pandemic (May 11, 2011) period than in the peak pandemic period (November 2009; Figure 5). However, some antibiotics did not show any appreciable decline in concentration (erythromycin, oxytetracycline and trimethoprim). It is unclear to what extent the persistence of these antibiotics in the river is related to the average river temperatures; it could be expected that higher river temperatures would facilitate the biotransformation of the antibiotics. The water temperature during the March 15, 2010 sampling was the lowest (8.2°C), followed by the four November sampling points (12.7, 9.7, 9.9 and 10.3°C respectively), while the water temperature in May 11, 2011 was the highest, 15.6°C. Evidence for an exponential relationship between the biodegradation rate and temperature suggest the temperature range in this study is likely to be an important variable in explaining the frequency and concentration of analytes recovered in different seasons [42], [43].

#### Decongestants

The detection of decongestants in Oxford WWTP effluent (notably in the lowest flowing period of the day), and not in the influent, suggests a degree of concentration within the WWTP. This is likely due to the relatively high lipophilicity of the decongestants (predicted log*P*: naphazoline 3.8, xylometazoline 5.2, and oxymetazoline 4.5; www.chemspider.com). High log*P* would facilitate partitioning of the decongestant onto suspended organic matter which is recycled in the Oxford WWTP. Notably, the trickling filter treatment of Benson WWTP also appeared to concentrate oxymetazoline, with negligible change in naphazoline and a significant decline in xylometazoline. Although all the decongestants are found in the WWTPs, they do not appear to persist in rivers, or if they do, they persist in an adsorbed state (i.e., to sediment), which was not measured as part of this study. Future studies will need to examine how representative this study is across a wider range of WWTPs and over a longer period of monitoring. Furthermore, future research should target different environmental matrices to ensure these pharmaceuticals are not accumulating in river sediment.

### Drug Use by Wastewater Epidemiology

#### Antiviral

The potential for wastewater epidemiology is highest when studying a recalcitrant, water soluble pollutant. This is one of the major reasons why the wastewater epidemiology approach has so much potential when applied to OC [34]. The physico-chemical benefits of OC are further enhanced because it is consumed as a prodrug. As such, improperly disposed Oseltamivir would be found in wastewater as the parent compound oseltamivir not OC. Hence, the difference between consumption and improper disposal can be relatively easily illuminated.

#### Antibiotics

To our knowledge the antibiotics within this study are not provided in a prodrug form, making estimates of their usage susceptible to misinterpretation owing to potential improper disposal. The recalcitrance of antibiotics is known to vary greatly in wastewater [44], [45] making it considerably more difficult to accurately predict environmental concentrations, as can been seen in Table 3. The variability in the recalcitrance of antibiotics in WWTPs and within the same WWTP over time and between differing WWTPs of different size and treatment technologies are likely among the main sources of error in the wastewater epidemiology approach when applied to antibiotics. Typically, the measured environmental load for each antibiotic (Table 3), was lower than the forward-calculated values from PCT or NHS BSA statistics, hence, the projected antibiotic users were frequently over estimated. Efforts to improve the wastewater epidemiology approach for antibiotics will need to address: (a) heterogeneity in the temporal distribution of prescriptions over time; (b) heterogeneity in the spatial distribution of prescriptions (across the UK) over time; (c) heterogeneity in *in vivo* and environmental stability of the antibiotic, including sewage pipes prior to reaching the WWTP inlet [46]; and (d) variability in compliance rate. It has been shown that the compliance rate for antibiotics can depend on the number of doses per day and age [47]–[49]. Further consideration should be given to (e) the sample size and sampling method. The relatively low number of pharmaceutical users in the two WWTP catchments, and the Benson WWTP catchment in particular, leaves model estimates of antibiotic users highly susceptible to systematic errors, as previously described [50]. The heterogeneity in the content of wastewater associated with low flush events, typical of low flow periods in the middle of the night, are a major factor influencing variations in analyte recovery over much of the sampling period [51]–[53]. This higher variability can be witnessed by the higher standard deviation in hourly measures of OC in Benson (433±472 ng/L) as compared to Oxford (358±60 ng/L). The Oxford and Benson sewer systems receive flow from a number of pumping stations that contribute to the mixing of discrete flushing events, however, the problems associated with sampling small populations would be more effectively alleviated with more intensive sampling (every 5–15 minutes) [50]. And finally, the wastewater epidemiology approach for antibiotics will likely be highly sensitive to (f) variability in environmental temperatures and precipitation, where low temperatures will likely retard biodegradation and high precipitation will dilute potentially inhibitory levels of drug while also resuspending sediment that can subsequently influence the drug’s fate.

#### Decongestants

The ability to predict decongestant users from measured concentrations in WWTP influent was constrained by the same systematic problems discussed earlier for the antibiotics, but might be further constrained by: 1) their apparent susceptibility to biodegradation; 2) their high rate of non-prescription use (i.e., over-the-counter), thereby hindering the acquisition of spatially and temporally resolved use data to confirm model projections; and 3) their more sporadic use pattern than antibiotics, the latter of which has a typical course of two to four tables per day for 7 to 10 days, whereas decongestants are only used as and when required. Given these many limitations, there was no ability to predict decongestant user numbers from measured environmental concentrations.

### Conclusions

In hindsight, the 2009 influenza A(H1N1) pdm09a virus generated a relatively small number of fatalities as compared to severe pandemics like the 1918 ‘Spanish flu’, which meant that the medical response was proportionately lower than would have been expected in a moderate or severe influenza pandemic. Hence, the potential negative effects to WWTP operation [13] and the environment proposed to occur in a moderate and severe pandemic [2], [14] were not reported. This study provides the first evidence that antibiotic and antiviral use was elevated during the pandemic. Theoretically, the antiviral recorded in the River Thames was of sufficient concentration to select for antiviral resistance in wildfowl [18], [54]. However, it remains to be demonstrated whether this had occurred.

There remains a great deal of uncertainty with regard to pharmaceutical use patterns during a pandemic, as a result of poor adherence to prescribed drugs [34] and the widespread use of over-the-counter medications. The focus on Oseltamivir here and in the literature is unlikely to reflect antiviral practices beyond 2020 owing to an increasing number of influenza antivirals in the pipeline [55]–[57]. However, for the time being, Oseltamivir remains one of the few antivirals within national stockpiles and as such, remains an important medical tool and potentially significant environmental pollutant [58]. Future influenza pandemics might, in fact, employ a combination therapy of two or more antivirals in an effort to combat resistance [59], [60].

Opportunities to ground truth model predictions for ‘black swan events’ such as influenza pandemics are, by definition, very rare (every 30 years), making this study conducted during the last influenza pandemic a unique window onto public health practice, human behavior, and drug adherence in the UK. It represents the first study to measure antibiotics and decongestants in influent and effluent and receiving rivers during a public health emergency, thereby establishing a baseline from which future modeling and risk assessments can be built in preparation for more severe public health emergencies.

## Supporting Information

File S1 **Figure S1.** Hourly total pharmaceutical load in Benson (A) and Oxford (B) WWTP inlet, for antibiotics (diamond), oseltamivir carboxylate (square), and decongestants (triangle) on November 10–11, 2009. No decongestants were detected in the Oxford WWTP inlet. **Figure S2.** Available dilution per capita per day at each of the River Thames sampling sites on each sampling occasion (November, 3, 10, 16, 24, 2009; March 15, 2010; May 11, 2011), dry weather flow (L/cap/d) and population. **Figure S3.** Atmospheric conditions on sampling days, including mean (max/min) temperature (°C) and precipitation (‘PP’; mm), on the sampling day, 24-hours and 48-hours ahead of the Thames River sampling occasion. **Figure S4.** Percentage of samples in Benson and Oxford WWTPs (n_max_ = 24) and the river Thames (n_max_ = 84; only November) from which analytes were found above their LOQ. Analytes arranged by least to most frequently found in the River Thames, from left to right. Brown = Benson WWTP influent; Blue = Benson WWTP effluent; Yellow = Oxford WWTP influent; Red = Oxford WWTP effluent; Black = River Thames. **Figure S5A.** Concentration of antibiotics and oseltamivir (ng/L) at all river sampling locations for each of the sampling dates: November 3, 2009. River sampling locations given sorted by population upstream with TC8 having the smallest population. **Figure S5B.** Concentration of antibiotics and oseltamivir (ng/L) at all river sampling locations for each of the sampling dates: November 10, 2009. River sampling locations given sorted by population upstream with TC8 having the smallest population. **Figure S5C.** Concentration of antibiotics and oseltamivir (ng/L) at all river sampling locations for each of the sampling dates: November 16, 2009. River sampling locations given sorted by population upstream with TC8 having the smallest population. **Figure S5D.** Concentration of antibiotics and oseltamivir (ng/L) at all river sampling locations for each of the sampling dates: November 24, 2009. River sampling locations given sorted by population upstream with TC8 having the smallest population. **Figure S5E.** Concentration of antibiotics and oseltamivir (ng/L) at all river sampling locations for each of the sampling dates: March 15, 2010. River sampling locations given sorted by population upstream with TC8 having the smallest population. **Figure S5F.** Concentration of antibiotics and oseltamivir (ng/L) at all river sampling locations for each of the sampling dates: May 11, 2011. River sampling locations given sorted by population upstream with TC8 having the smallest population. **Table S1.** NHS BSA statistics on drug use in England from 2007–8. Predicted mass of drug used per day per WWTP catchment, using winter adjusted values, where the annual prescription rate was adjusted by a factor of 9.375% higher than the annual average rate (see text for details). Benson drug use rate was adjusted by a factor of 0.389 (the estimated fraction of the local PCT population within the Benson WWTP catchment; see text for details). na = data not available. **Table S2.** Literature review of oseltamivir in WWTP and rivers. **Table S3.** Liquid chromatography-tandem mass-spectrometry (LC-MS/MS) method parameters of study analytes.
(DOCX)
