# Supplementary material for: Intra- and Inter-Pandemic Variations of Antiviral, Antibiotics and Decongestants in Wastewater Treatment Plants and Receiving Rivers
Source: PLoS One. 2014 Sep 25;9(9):e108621. doi: 10.1371/journal.pone.0108621 (PMC4177917; doi:10.1371/journal.pone.0108621)
Supplement: File S1 — Figure S1. Hourly total pharmaceutical load in Benson (A) and Oxford (B) WWTP inlet, for antibiotics (diamond), oseltamivir carboxylate (square), and decongestants (triangle) on November 10–11, 2009. No decongestants were detected in the Oxford WWTP inlet. Figure S2. Available dilution per capita per day at each of the River Thames sampling sites on each sampling occasion (November, 3, 10, 16, 24, 2009; March 15, 2010; May 11, 2011), dry weather flow (L/cap/d) and population. Figure S3. Atmospheric conditions on sampling days, including mean (max/min) temperature (°C) and precipitation (‘PP’; mm), on the sampling day, 24-hours and 48-hours ahead of the Thames River sampling occasion. Figure S4. Percentage of samples in Benson and Oxford WWTPs (nmax = 24) and the river Thames (nmax = 84; only November) from which analytes were found above their LOQ. Analytes arranged by least to most frequently found in the River Thames, from left to right. Brown = Benson WWTP influent; Blue = Benson WWTP effluent; Yellow = Oxford WWTP influent; Red = Oxford WWTP effluent; Black = River Thames. Figure S5A. Concentration of antibiotics and oseltamivir (ng/L) at all river sampling locations for each of the sampling dates: November 3, 2009. River sampling locations given sorted by population upstream with TC8 having the smallest population. Figure S5B. Concentration of antibiotics and oseltamivir (ng/L) at all river sampling locations for each of the sampling dates: November 10, 2009. River sampling locations given sorted by population upstream with TC8 having the smallest population. Figure S5C. Concentration of antibiotics and oseltamivir (ng/L) at all river sampling locations for each of the sampling dates: November 16, 2009. River sampling locations given sorted by population upstream with TC8 having the smallest population. Figure S5D. Concentration of antibiotics and oseltamivir (ng/L) at all river sampling locations for each of the sampling dates: November 24, 2009. River samplin [file pone.0108621.s001.docx]

**Supporting Information 1.**

Figure S1. Hourly total pharmaceutical load in Benson (A) and Oxford (B) WWTP inlet, for antibiotics (diamond), oseltamivir carboxylate (square), and decongestants (triangle) on November 10-11, 2009. No decongestants were detected in the Oxford WWTP inlet.

| A | B |
| --- | --- |

Figure S2. Available dilution per capita per day at each of the River Thames sampling sites on each sampling occasion (November, 3, 10, 16, 24, 2009; March 15, 2010; May 11, 2011), dry weather flow (L/cap/d) and population

Figure S3. Atmospheric conditions on sampling days, including mean (max/min) temperature (°C) and precipitation (‘PP’; mm), on the sampling day, 24-hours and 48-hours ahead of the Thames River sampling occasion.

Figure S4. Percentage of samples in Benson and Oxford WWTPs (n_max_=24) and the river Thames (n_max_=84; only November) from which analytes were found above their LOQ. Analytes arranged by least to most frequently found in the River Thames, from left to right. Brown = Benson WWTP influent; Blue = Benson WWTP effluent; Yellow = Oxford WWTP influent; Red = Oxford WWTP effluent; Black = River Thames.

Figure S5A. Concentration of antibiotics and oseltamivir (ng/L) at all river sampling locations for each of the sampling dates: November 3, 2009. River sampling locations given sorted by population upstream with TC8 having the smallest population.

A

Figure S5B. Concentration of antibiotics and oseltamivir (ng/L) at all river sampling locations for each of the sampling dates: November 10, 2009. River sampling locations given sorted by population upstream with TC8 having the smallest population.

B

Figure S5C. Concentration of antibiotics and oseltamivir (ng/L) at all river sampling locations for each of the sampling dates: November 16, 2009. River sampling locations given sorted by population upstream with TC8 having the smallest population.

C

Figure S5D. Concentration of antibiotics and oseltamivir (ng/L) at all river sampling locations for each of the sampling dates: November 24, 2009. River sampling locations given sorted by population upstream with TC8 having the smallest population.

D

Figure S5E. Concentration of antibiotics and oseltamivir (ng/L) at all river sampling locations for each of the sampling dates: March 15, 2010. River sampling locations given sorted by population upstream with TC8 having the smallest population.

E

Figure S5F. Concentration of antibiotics and oseltamivir (ng/L) at all river sampling locations for each of the sampling dates: May 11, 2011. River sampling locations given sorted by population upstream with TC8 having the smallest population.

F

Table S1. NHS BSA statistics on drug use in England from 2007-8. Predicted mass of drug used per day per WWTP catchment, using winter adjusted values, where the annual prescription rate was adjusted by a factor of 9.375% higher than the annual average rate (see text for details). Benson drug use rate was adjusted by a factor of 0.389 (the estimated fraction of the local PCT population within the Benson WWTP catchment; see text for details). na = data not available

|  | |  |  |  | |  | Winter adjusted  g drug/capita/d per WWTP catchment | |
| --- | --- | --- | --- | --- | --- | --- | --- | --- |
|  | TOTAL ADQs in England 2007-8 (x10^6^) | ADQ  (g) | Average Daily Users in England | Average Daily Users (winter normalised) | Prescriptions per 100,000 persons in England per day |  | Benson | Oxford |
| Trimethoprim | 19.65 | 0.4 | 53836 | 58883 | 116 |  | 1.12 | 96.06 |
| Oxytetracycline | 3.750 | 1 | 10274 | 11237 | 22.0 |  | 0.19 | 16.04 |
| Ofloxacin | 1.250 | 0.4 | 3425 | 3746 | 7.34 |  | 0.01 | 0.48 |
| Norfloxacin | 0.100 | 0.8 | 274 | 300 | 0.588 |  | 0.01 | 0.88 |
| Oseltamivir | na | 0.15 |  |  |  |  |  |  |
| Ciprofloxacin | 11.50 | 0.75 | 31507 | 34461 | 67.6 |  | 1.23 | 105.41 |
| Naphazoline | na | 0.4 |  |  |  |  |  |  |
| Azithromycin | 1.250 | 0.5 | 3425 | 3746 | 7.3 |  | 0.08 | 6.49 |
| Cefotaxime | na | 4 |  |  |  |  |  |  |
| Doxycycline | 3.000 | 0.1 | 8219 | 8990 | 17.6 |  | 0.03 | 2.93 |
| Sulfamethoxazole | 0.900 | 0.8 | 2466 | 2697 | 5.29 |  | 0.10 | 8.80 |
| Oxymetazoline | na | 0.4 |  |  |  |  |  |  |
| Erythromycin | 27.50 | 1 | 75342 | 82406 | 162 |  | 3.92 | 336.09 |
| Xylometazoline | na | 0.8 |  |  |  |  |  |  |
| Clarithromycin | 11.25 | 0.5 | 30822 | 33711 | 66.1 |  | 0.44 | 37.81 |

Table S2. Literature review of oseltamivir in WWTP and rivers.

| Location | WWTP (ng/L) | River (ng/L) | Inter-/Intra- Pandemic | Reference |
| --- | --- | --- | --- | --- |
| England | Benson influent:  433 ± 472 (max = 2070)  Benson effluent:  208 ± 40 (max = 287)  Oxford influent:  350 ± 59 (max = 550)  Oxford effluent:  358 ± 60 (max = 463) | 49 ± 40 (max=193) | Intra- | (this study) |
| China | <11 | < 11 | Inter- | ([Peng et al. 2014](#_ENREF_57)) |
| Japan | 29-650 | 7.3-411 | Inter- | ([Azuma et al. 2013](#_ENREF_1)) |
| Japan | 130-482 | 68-557 | Inter- | ([Takanami et al. 2012](#_ENREF_35)) |
| Norway | <3-1450 |  | Intra- | ([Leknes et al. 2012](#_ENREF_36)) |
| Japan | 293 | 193 | Inter- | ([Ghosh et al. 2010a](#_ENREF_37)) |
| Spain |  | 22-50 | Intra- | ([Gonçalves et al. 2011](#_ENREF_58)) |
| Japan | 177-827 | 60-288 | Intra- | ([Azuma et al. 2012](#_ENREF_34)) |
| Japan |  | 10-864 | Intra- | ([Takanami et al. 2010](#_ENREF_40)) |
| Germany | 12 – 43 | 0.6-24 | Inter- | ([Prasse et al. 2010](#_ENREF_33)) |
| Japan | ~10-293 | ~7-293 | Inter- | ([Ghosh et al. 2010b](#_ENREF_38)) |
| Japan | ~370-400  ~150-460 |  | Inter-  Intra- | ([Ghosh et al. 2010a](#_ENREF_37)) |
| Japan |  | 2-58 | Inter- | ([Soderstrom et al. 2009](#_ENREF_59)) |

Table S3. Liquid chromatography-tandem mass-spectrometry (LC-MS/MS) method parameters of study analytes

| Study analytes | Internal standard used | Precursor → product | Collision energy | Tube lens | Retention time |
| --- | --- | --- | --- | --- | --- |
|  |  | (m/z) | (V) | (V) | (min) |
| *Antibiotics* |  |  |  |  |  |
| Azithromycin | ^13^C_2_-ERY | 749.40 → 158.20 | 37 | 148 | 6.45 |
|  |  | 749.40 → 591.60 | 28 | 148 |  |
| Cefotaxime | ^13^C_6_-SUL | 455.90 → 396.10 | 5 | 106 | 6.21 |
|  |  | 455.90 → 125.10 | 31 | 106 |  |
| Ciprofloxacin | ^13^C_3_-CIP | 332.00 → 231.10 | 35 | 117 | 5.76 |
|  |  | 332.00 → 288.20 | 16 | 117 |  |
| Claritromycine | ^13^C_2_-ERY | 748.40 → 158.10 | 27 | 156 | 7.88 |
|  |  | 748.40 → 590.50 | 17 | 156 |  |
| Doxycycline | ^13^C_2_-TRI | 445.00 → 428.30 | 17 | 107 | 6.83 |
|  |  | 445.00 → 200.90 | 27 | 107 |  |
| Erythromycin | ^13^C_2_-ERY | 734.30 → 576.60 | 19 | 154 | 7.30 |
|  |  | 734.30 → 158.10 | 29 | 154 |  |
| Norfloxacin | ^13^C_3_-CIP | 320.00 → 302.10 | 20 | 114 | 5.71 |
|  |  | 320.00 → 233.10 | 23 | 114 |  |
| Ofloxacin | ^13^C_3_-CIP | 362.10 → 318.20 | 17 | 138 | 5.68 |
|  |  | 362.10 → 261.10 | 25 | 138 |  |
| Oxytetracycline | ^13^C_2_-TRI | 461.00 → 426.20 | 18 | 130 | 5.60 |
|  |  | 461.00 → 443.20 | 13 | 130 |  |
| Sulfamethoxazole | ^13^C_6_-SUL | 254.00 → 108.20 | 22 | 108 | 6.90 |
|  |  | 254.00 → 156.00 | 15 | 108 |  |
| Trimetoprim | ^13^C_2_-TRI | 291.00 → 230.10 | 23 | 106 | 5.44 |
|  |  | 291.00 → 123.20 | 25 | 106 |  |
| *Decongestants* |  |  |  |  |  |
| Naphazoline | ^13^C_2_-TRI | 211.10 → 141.20 | 33 | 73 | 5.99 |
|  |  | 211.00 → 44.50 | 23 | 73 |  |
| Oxymetazoline | ^13^C_2_-ERY | 261.10 → 205.20 | 25 | 93 | 7.21 |
|  |  | 261.10 → 135.20 | 33 | 93 |  |
| Xylometazoline | ^13^C_2_-ERY | 245.10 → 189.20 | 26 | 91 | 7.60 |
|  |  | 245.10 → 119.30 | 35 | 91 |  |
| *Antiviral* |  |  |  |  |  |
| Oseltamivir carboxylate (^2^H-OC) | - | 285.00 → 138.10 | 20 | 100 | 5.70 |
|  |  | 285.00 → 120.20 | 30 | 100 |  |
| *Internal standards* | | | | |  |
| ^13^C_2_-Trimetoprim (^13^C_2_-TRI) | - | 294.10 → 233.20 | 22 | 101 | 5.44 |
|  |  | 294.10 → 126.20 | 24 | 101 |  |
| ^2^H-Oseltamivir carboxylate (^2^H-OC) | - | 288.00 → 139.00 | 19 | 100 | 5.70 |
|  |  | 288.00 → 95.00 | 30 | 100 |  |
| ^13^C_3_-Ciprofloxacin (^13^C_3_-CIP) | - | 336.00 → 291.00 | 18 | 106 | 5.78 |
| ^13^C_6_-Sulfamethoxazole ( ^13^C_6_-SUL) | - | 260.00 → 162.10 | 15 | 97 | 6.89 |
|  |  | 260.00 → 114.20 | 23 | 97 |  |
| ^13^C_2_- Erythromycin (^13^C_2_-ERY) | - | 736.40 → 160.10 | 17 | 129 | 7.30 |
|  |  | 736.40 → 578.60 | 29 | 129 |  |
